# Highest Orthodontia

**Published:** 1905-06

**Authors:** 


### Reviews of Dental Literature.

Highest Orthodontia. 1. Facial Beauty; 2. Dental Antagonism. By John Nutting Farrar, M.D., D.D.S., New York City.[1] (Continued from page 299.)

Irregularity is seldom found in very wide jaws, but is often found in narrow jaws; yet the dental arches may be too wide. I have seen cases of regular teeth where the antagonism had forced the upper halves of the arch so far outward as to cause not only separation of the two halves of the jaw-bone, but also separation of the central incisors. The teeth of some upper and lower arches react so detrimentally upon each other that the arches not only become larger and larger, but the spaces between the teeth become wider and wider.

Both of the dental arches can be widened, but whether both need to be widened depends upon somewhat rare circumstances. It may also be said with considerable degree of truth, that the progress in operations for regulating teeth depends upon the amount of osseous tissue that must be absorbed, bent, or otherwise made to yield before the tooth or teeth to be moved. The conformation of the upper and lower jaws differ widely; so do the corresponding parts of the alveolar ridges differ, and the operations for widening must in a measure differ correspondingly. Before a body of intel-

---

[1] Lecture delivered before the New York Dental College Alumni, January 18, 1905.

ligent professional men like this it may be unnecessary to refer to anatomy of the jaws, but in order to make myself clearly understood, let us review the subject a moment.

While the anterior half of the lower jaw is narrower than the anterior half of the upper, the posterior part of the lower is wider than that of the upper. So do the corresponding parts of the two alveolar ridges that support the teeth also differ. While the inner, or lingual, wall of the upper ridge is much more massive than the outer wall, the entire inner, or lingual, wall of the lower is thin. But while the outer wall of all the upper sockets is thin, it is only the anterior outer half of the lower sockets that is thin. The outer wall of the posterior, or molar region is very thick and massive. Because of the thinness of the outer wall of the upper alveolar ridge, the operation for widening the entire length of the (upper) arch is comparatively easy, and when completed the teeth are easily retained in their new positions, provided they antagonize upon firm lower teeth. In regard to the lower arch, the widening of the anterior part is also comparatively easy, as far back as the molars, but because of the great thickness of the buccal side of the molar region, the widening of this section is very slow and tedious. While the thinness of the upper alveolar ridge permits of its teeth being easily moved outward throughout its length, the formation of the outer rear half of the lower is so massive that it is difficult if possible to bend outward. Indeed, these posterior parts are so thick that, if the teeth are to be moved outward, the work must be mainly done through absorption of the bone. But as the apices of the roots of these molar teeth, may, by leverage force, be liable to protrude through the lingual walls of the sockets, care should be exercised if the attempt be made to widen. While, however, these difficulties are true of the lower outer molar region, the teeth anterior to the molars, because of thinness of these alveolar walls, can be moved easily either way.

There are cases of lower arches that need widening, and should be widened, but they are few compared with the number of upper cases that need widening. But whether the upper arch should be widened by always moving the teeth against the sockets, or widened by opening the median suture (excepting the question of antagonism), is not within the province of this essay upon Beauty *vs.* Antagonism.

It is fortunate that in a great majority of cases the lower molars do not need to be moved, nor is it often necessary to move the side teeth anterior to these molars, unless to even an irregular line. Of the few lower teeth that need moving, most of them are the incisors, cuspids, and the second bicuspids; nor is it often necessary to widen the arch to accomplish the best results, as the extraction of an incisor (generally a central) may be more advisable, if by so doing no space be left; these incisors are so nearly of the same size and form, the loss of one is scarcely noticeable. This operation is especially applicable to cases having narrow heads and faces. In normal cases the outer cusps of the upper side teeth overhang closely the outer surface of the lower. The value of this arrangement for the greater bearing upon the fulcral line, along the lower ridge of the jaw, and less upon the lingual side of the ridge and against the thinner wall of the lower sockets, is apparent to the experienced regulator.

While a lower dental arch that needs widening or enlarging is seldom found, there are many upper arches that do need to be widened or enlarged, or both, in order to antagonize properly with the lower teeth. There are also, as before implied, many lower cases in which it is necessary to move one or two side teeth in order to even the line, and there are many cases where the anterior lower teeth are irregular, and need evening in order to properly round out the contour of the lips; the upper as well as the lower.

The small percentage of cases where the lower arch needs widening is fortunate to both operator and patient, because of the avoidance of difficulty of permanently retaining several moved lower side teeth in place sufficiently long to prevent them from becoming driven out of place by antagonism with the upper; more difficult is this than to permanently fix regulated upper teeth. If the widening of the upper arch to make room for the alignment of jumbled teeth, and secure proper antagonism in a thin face and narrow forehead, would injure facial beauty, it should seldom be attempted. But right here is room for careful judgment of the best kind. Different cases may require corresponding differences in the degree of widening.

There is more glory in one entirely successful operation, than in many partially successful operations. One complete success is a permanent evidence of excellence, while partial successes are standing evidences against the operator. To properly move teeth into their proper places is evidence of merit, but to move teeth so that they will not only permanently stay, but cause great beauty of the face, is *proof* of mastership.

The olden time dentists (able in some directions) did not seem to recognize high merit in correction of irregular teeth. Their principal idea of an operation appeared to lie along the line of "evening the row" of teeth, without much regard to antagonism, and with no idea whatever of the value of artistic moulding of the face, further than would result from simply the alignment of the teeth; even this was not often attempted in difficult cases.

When the teeth were greatly jumbled, overlapped, overcrowded, they thought extraction of some of them (and it did not matter much which tooth or teeth) was the only proper way to make room. It is not necessary to say that this plan without artistic aims, without ability to prognosticate with certainty the results, must have led to more or less incongruity, if not total failure. After the days of Delabarre (1826) and Maury (1828) there came a reaction, and that which was regarded as "normal antagonism," but without much idea of that which constitutes proper antagonism, was looked upon as the panacea for all forms of irregularities. It was not taught that antagonism as generally found in nature, in the human race, was seldom perfect antagonism; that it was so rare that in mixed races as found along the border between different nations, and in the United States, only about one per cent. of all is any way near perfect. The normal antagonism hypothesis, as then regarded, had its career, however. This was followed by the hypothesis that all irregular upper teeth should be evened by widening the arch, disregarding the lower. Then followed the hypothesis of normal antagonism by widening both dental arches, if necessary, to even all the irregular teeth. But while the upper arch was often widened, even beyond the antagonizing line of the lower teeth, but few attempts were made to widen both arches at the same time. This plan of so-called "normal" was followed by the hypothesis of "perfect" antagonism, which is still taught somewhat.

In about 1875 the essayist began to express views that although each plan had some points of value, none of the hypotheses previously in vogue could be regarded as an improvement, *par excellence,* over those advocated in earlier days, and that the question of antagonism really was but the alphabet of the subject of this science and art. I then thought, and still think, and have so taught for many years, that the fundamental steps, and all the way up the ladder of knowledge in this line, the aim of the regulator of teeth should be first the accomplishment of the highest degree of beauty of the patient's face, and, as a rule, whatever is necessary to aid in securing this highest result is legitimate and proper. To maintain this highest facial improvement, it may require nearly perfect antagonism; but if perfect antagonism would injure the accomplishment of the highest degree of facial beauty, the attempt at perfection of antagonism may become questionable. Beauty should be carried as high as possible under the circumstances of the case in hand. The great desideratum is beauty with efficient antagonism; not necessarily perfection of antagonism. In other words, the dentist should accomplish as near perfection of antagonism as possible, consistent with the highest degree of facial beauty. But to bring about all this excellence may require a higher grade of mechanical aid than was advocated in the past (1874–75). To meet this lacking was my object in publishing, free to all, many mechanical inventions in many papers upon the subject in the *Dental Cosmos* and various other journals in America and Europe, and later published in book form.

It may here be proper to mention the evil effects of overwidening the arch. By the term overwidening is meant the unnecessary widening. To overwiden the upper arch, carrying the side teeth beyond and outside of the lower teeth, leaving the upper teeth without proper antagonizing restraint and difficult to be safely retained, is questionable practice. To overwiden the upper arch and, at or near the same time, widen the lower arch to match the upper teeth, places the whole case in a dangerous condition, because it is difficult to safely maintain the antagonism between the two unstable arches, unless harnessed in place by retainers for a long time. I do not refer to the very few cases where the opposing arch creeps along with the one being widened. Some of the worst forms of malantagonism and failures that I ever saw were the results of overwidening the upper arch, followed closely by the widening of the lower arch in the attempt at perfection of antagonism.

The reactive tendency of tooth sockets, together with the various directions of force upon the teeth by the lingual, buccal, and labial muscles during the act of swallowing food, and several times every minute by similar action of these muscles while swallowing saliva, causes a combination of forces upon the loosened teeth that tends to prevent a steady retentive disposition, and then one or several teeth, being thus left in unstable conditions, will drift back more or less toward their original places. Nor does the use of retaining mechanisms, as usually made and applied, suffice to overcome these evil tendencies, unless worn too long for the best interests of the tissues of the teeth. If, however, small retaining mechanisms can be cemented upon the teeth so as to be safely left for a long time, they may be advisable. The use of large retainers, that must be worn when both arches are widened at the same time, are generally dangerous collectors of food débris, consequently should not be used except upon very intelligent and careful patients, who will be sure to keep these retainers and the teeth clean. The question of retaining the teeth in place sufficiently long for them to firmly set and remain so, is sometimes more difficult to answer than the question, How shall teeth be moved? It is not difficult to move teeth, but to move them properly, and so that they will stay fixed, is not always easy, and to regulate teeth so as to mould the face to its highest possibilities in beauty is still more difficult, and a comparative examination of photographs of a "perfect case," before and after five years' tests, that shows no change, is not only difficult but very rare. I will say, however, in regard to the latter phase, that it is not always the regulator's fault if teeth do not remain corrected, for after repeated warnings to the patients to return to the operator to have him watch the cases, generally little or no attention is given to the generous request. This indifference is not confined to patients after completion of cases, but is often noticeable in failure to keep appointments during the regulating process. Punctuality is as necessary in this line of work as in other lines.

When a tooth is moved its firm socket-hold becomes more or less weakened, and the tooth is easily artificially retained in place, but when several teeth are moved, and are loose, the difficulty is greater, especially so if the sockets have a tendency to be loculitic (pyorrhœic). When this combination of weaknesses exists, the loose teeth immediately begin playing back and forth, by force of the muscles of the cheeks and tongue, which, together with the disturbing action, from the wrongly inclined bearings of the cusps of the opposing teeth (in the act of swallowing), renders sufficient permanency for new tissue to form and mature almost impossible. The old school idea, that antagonism is the highest single phase of dentistry, has a degree of truth, however, so far as it goes, just as the alphabet is the basal principle of literary education, but the alphabet of this subject (antagonism) is only the beginning of the highest education in the science and art of correction of irregularities of the teeth, and the remodelling of the face to the highest possibilities.

The proper relation of the teeth, and the proper form of the teeth, and the proper form of the arches of the thin face and narrow head, are different from the proper relation of the teeth and the form of the arches necessary for the wide face. Not only is the poise of the teeth of the upper and the lower jaw different, but the position of the teeth should be maintained by firm antagonism (not rickety). Again, I find by forty years of experience that by far the larger number of cases of protruding (upper) teeth require the dental arch to be shortened, and do not need to be widened. That while to reverse this act (widening) would weaken facial expressions, and would not lead to the highest degree of beauty of the face, the judicious shortening of the arch does not disturb antagonism the least, and in every way improves the case the most.

(To be continued.)

